# Novel Techniques, Biomarkers and Molecular Targets to Address Cardiometabolic Diseases

**DOI:** 10.3390/jcm13102883

**Published:** 2024-05-14

**Authors:** Valerio Di Fiore, Federica Cappelli, Lavinia Del Punta, Nicolò De Biase, Silvia Armenia, Davide Maremmani, Tommaso Lomonaco, Denise Biagini, Alessio Lenzi, Matteo Mazzola, Domenico Tricò, Stefano Masi, Alessandro Mengozzi, Nicola Riccardo Pugliese

**Affiliations:** 1 Department of Clinical and Experimental Medicine, University of Pisa, Via Roma 67, 56124 Pisa, Italyfederica.cappelli@med.unipi.it (F.C.);; 2 Department of Chemistry and Industrial Chemistry, University of Pisa, Via Giuseppe Moruzzi 13, 56124 Pisa, Italy; tommaso.lomonaco@unipi.it (T.L.);; 3 Department of Surgical, Medical and Molecular Pathology and Critical Care Medicine, University of Pisa, Via Paradisa 2, 56124 Pisa, Italy

**Keywords:** cardiometabolic desease, arterial hypertension, type 2 diabetes mellitus, heart failure, novel markers

## Abstract

Cardiometabolic diseases (CMDs) are interrelated and multifactorial conditions, including arterial hypertension, type 2 diabetes, heart failure, coronary artery disease, and stroke. Due to the burden of cardiovascular morbidity and mortality associated with CMDs’ increasing prevalence, there is a critical need for novel diagnostic and therapeutic strategies in their management. In clinical practice, innovative methods such as epicardial adipose tissue evaluation, ventricular–arterial coupling, and exercise tolerance studies could help to elucidate the multifaceted mechanisms associated with CMDs. Similarly, epigenetic changes involving noncoding RNAs, chromatin modulation, and cellular senescence could represent both novel biomarkers and targets for CMDs. Despite the promising data available, significant challenges remain in translating basic research findings into clinical practice, highlighting the need for further investigation into the complex pathophysiology underlying CMDs.

## 1. Introduction

**Figure 1:**
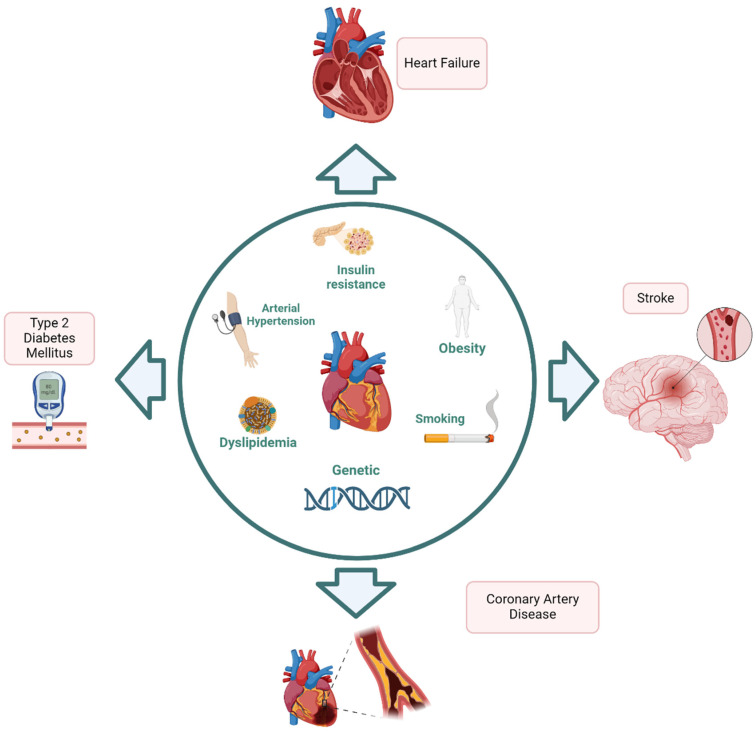
Pathophysiology of cardiometabolic diseases.

Cardiometabolic diseases (CMDs) are several interrelated and multifactorial conditions, including arterial hypertension (AH), type 2 diabetes (T2D), heart failure (HF), coronary artery disease (CAD) and stroke (Figure 1). The combined presence of many CMDs is associated with higher mortality risk [1]. Particularly, cardiometabolic multimorbidity, defined as the presence of ≥2 cardiometabolic diseases, represents an issue for global public health [2]. Patients over 60 years of age with concomitant T2D, acute myocardial infarction, and/or stroke seem to have a reduced life expectancy by 15 years [2]. The concomitant presence of several cardiovascular diseases is caused by common cardiovascular risk factors such as AH, dyslipidaemia, and T2D but also by mutually affecting pathophysiological mechanisms such as atherosclerosis, thromboembolic activation, haemodynamic failure, and neuroendocrine feedback activation [3]. AH is a major preventable cause of atherosclerotic cardiovascular diseases and death from all causes [4], being responsible for the high burden of cardiovascular morbidity and mortality and all-cause mortality [4]. AH, together with other metabolic abnormalities such as abdominal (or visceral) obesity, dyslipidaemia, glucose intolerance, insulin resistance, and hyperuricemia, can lead to metabolic syndrome, which is associated with high cardiovascular risk [5]. Nevertheless, cardiometabolism is still largely unstudied, partially due to the extent of the pathophysiological pathways involved [6,7]. Identifying novel biomarkers and molecular targets is crucial for improving diagnosis, prognosis, and treatment. This review investigates the new multimodal approaches spreading in evaluating CMDs, along with novel biomarkers and molecular targets.

### 1.1. Novel Techniques and Biomarkers in the Clinical Setting

HF is a clinical syndrome due to structural and functional abnormalities of the heart that result in elevated intracardiac pressures and/or inadequate cardiac output, characterised by cardinal symptoms and signs [8]. Among CMDs, HF drives cardiovascular disability and mortality, and due to the complexity of its pathophysiology, involving several neurohormonal and chemical pathways, it can be considered a prototype of CMDs. HF is commonly classified based on left ventricular ejection fraction (LVEF) [8]. Notably, HF with reduced LVEF (HFrEF; LVEF < 50%) and mildly reduced EF (HFmrEF; LVEF 40–49%) are associated with high catabolic activity compared with heart failure with preserved LVEF (HFpEF; LVEF ≥ 40%), which in turn is characterised by metabolic/inflammatory derangements [7,9]. Hence, HFpEF incidence continues to rise with increasing ageing, obesity, sedentariness, and CMDs [10]. Novel diagnostic methods and markers have been proposed and might be applied to evaluating CMDs, including HFpEF [6] (Table 1).

#### 1.1.1. Epicardial Adipose Tissue

Epicardial adipose tissue (EAT) is commonly found in the atrioventricular and interventricular grooves and along the free walls of the atria, between the visceral epicardium and the outer myocardial surface [11]. EAT has either favourable or unfavourable effects on the cardiovascular system homeostasis. On one side, it protects the heart against high fatty acid levels and provides a local energy source at times of high demand [12]. Conversely, EAT releases bioactive adipokines, such as chemerin, resistin, serglycin, and intelectin 1 (also known as omentin 1), that promote harmful coronary artery and myocardial changes [11]. Indeed, increased echocardiographic EAT thickness is associated with coronary artery disease, subclinical atherosclerosis, insulin resistance, and metabolic syndrome. Obesity promotes systemic inflammation by driving adipogenesis, probably due to an adaptive mechanism promoting proinflammatory fatty acid deposition in cells other than adipocytes [13]. Similarly, increased EAT could express the so-called inflammatory-metabolic phenotype of HFpEF [14,15]. In such patients, the epicardium becomes a site of deranged adipogenesis, producing proinflammatory adipokines that can cause atrial and ventricular fibrosis [13]. Moreover, EAT plays a mechanical role as it exerts direct compression on the myocardium within the closed pericardial sac [16], leading to enhanced pericardial restraint [15]. Contrariwise, EAT thickness is reduced in patients with HFrEF, probably as an expression of cardiac cachexia, characterised by enhanced lipolytic activity and a metabolic shift towards catabolism [15]. The quantification of EAT thickness is a helpful diagnostic tool for clinicians. In a clinical setting, visceral fat is typically measured by surrogate markers, such as waist circumference alone or the ratio of waist circumference to hip circumference. EAT could be accurately measured using cardiac magnetic resonance or cardiac computerised tomography [12], but they are expensive methods unsuited to clinical practice. Therefore, EAT thickness can be assessed with two-dimensional echocardiography: standard parasternal long-axis and short-axis views permit the most accurate measurement of epicardial fat thickness on the right ventricle, with optimal cursor beam orientation in each view [12]. EAT thickness can be identified as the echo-free space between the outer wall of the right ventricle and the visceral pericardium at end-systole, averaged from two cardiac cycles [15]. In the future, it would be challenging to estimate also EAT activity through the measurement of the changes in its mRNA expression and proinflammatory cytokine levels, such as tumour necrosis factor-α, resistin, monocyte chemoattractant protein-1 and adiponectin [17].

#### 1.1.2. Congestion

Congestion is the presence of signs and symptoms of extracellular fluid accumulation [18] that results from increased cardiac filling pressures [19]. The latter also induce natriuretic peptide release, such as B-type natriuretic peptide (BNP) and N-terminal pro-B-type natriuretic peptide (NT-proBNP). Indeed, BNP and) are released by cardiomyocytes in response to volume and pressure overload, and their increased levels correlate with poor prognosis [14]. NT-proBNP shows elevated concentrations in most forms of HF, making it a crucial diagnostic marker for HF in many clinical settings, especially when the diagnosis is uncertain [14]. Also, increased levels of natriuretic peptides correlate with poor prognosis [14], while reducing NT-proBNP is associated with decreased HF hospitalisation rates and CV death [20]. However, in some HF patients, particularly those with HFpEF, natriuretic peptide levels might be lower compared to individuals with HFrEF, although typically, these levels are still above those found in people without heart failure [17]. In patients with HFpEF, determinants of natriuretic peptide elevation in HFpER are less well understood: LV chamber size is generally smaller, which may explain lower wall stress, although the pathophysiology and natural history of this phenotype remain unclear [21]. The gold standard for cardiac filling pressures is the direct measurement of right atrial pressure and pulmonary capillary wedge pressure (PCWP) through cardiac catheterisation [22]. Due to its invasive nature, catheterisation has limitations in routine use, and the detection of congestion remains a diagnostic challenge due to the late onset of symptoms and signs [23]. Ultrasound (US) may play a role in the non-invasive estimation of PCWP [24]. However, these are indirect measurements, while US can provide a more direct assessment of congestion, even when it is only subclinical [25]. Over the last decades, new ultrasound methods have been developed, enabling earlier and more accurate diagnosis and facilitating treatment strategies. Traditionally, increased central venous pressure can be estimated by measuring the inferior vena cava (IVC) and/or jugular vein distention [25]. More recently, Doppler-derived renal venous flow (RVF) has been proposed as an early marker of renal congestion, demonstrating a significant prognostic role in HF, irrespective of LVEF [19]. Similarly, lung ultrasound can quantify extravascular lung fluid by counting the artefacts known as B-lines [22]. In patients with HF, the simultaneous assessment of pulmonary, venous, and kidney congestion by US improves the identification of sub-clinical congestion associated with poor outcomes. Intriguingly, US congestion is also detectable in patients without HF but with other CMDs such as AH and T2D [22]. The addition of other biomarkers such as atrial natriuretic peptide (ANP), high-sensitivity cardiac troponin and soluble suppression of tumorigenicity 2 protein (sST2), a biomarker of cardiac stress encoded by the interleukin 1 receptor-like 1 gene, may help to better understand underlying pathophysiology and natural history of CMDs, by predicting the trajectory of subclinical damage like cardiac remodelling [26].

#### 1.1.3. Ventricular–Arterial Coupling

As arterial load and stiffness are related to LV function, mass, and myocardial deformation, the evaluation of the relationship between the LV and the arterial system, also known as ventricular–arterial coupling (VAC), has shown an independent diagnostic and prognostic value in different CMDs [27,28]. Despite its complexity, several invasive and non-invasive methods have been developed to measure VAC. The pressure–volume loops assessed by cardiac catheterisation allow the understanding of the heart–vessel coupling, as it constantly changes to match ventricular end-systolic and arterial elastances. Considering the left ventricle as a closed volume, end-systolic elastance (Ees) represents the necessary intracavitary pressure to increase its volume by one unit, while the end-systolic pressure to stroke volume (SV) ratio is termed effective arterial elastance (Ea). Stroke work generation is maximal when the Ea/Ees ratio equals 1, while cardiac efficiency is maximal when the Ea/Ees ratio equals 0.5 [27]. Echocardiography can non-invasively approximate Ea/Ees and its components but at the cost of complexity or poor reproducibility [27].

Arterial stiffness is a pivotal element in VAC derangement and is also linked with ageing and CMDs. Aortic characteristic impedance, aortic distensibility, and beta stiffness index have been proposed as markers of arterial function. Still, they need significant expertise for estimation, and limited and prognostic data are available [27]. Tonometry-derived carotid–femoral pulse wave velocity (cf-PWV) is the gold standard for measuring central arterial stiffness [27]. In hypertensive heart disease, high values of cf-PWV are associated with reduced aerobic exercise capacity [29]. The ratio between cf-PWV and myocardial deformation (global longitudinal strain [GLS]) has been proposed to characterise VAC [30] and has been shown to predict mortality following hospitalisation for HFpEF [31]. Nevertheless, cf-PWV has several limitations. First, it is time-consuming, as it usually requires sequential recordings of the carotid and femoral signals triggered by the electrocardiogram (foot-to-foot method) [32]. Moreover, cf-PWV ignores the ascending aorta and aortic arch stiffness, the measurement of surface distance needs a correction factor to adjust for overestimating aortic length (particularly in elderly patients), and pulsed wave detection can be challenging in some patients (e.g., obese patients) [32]. To overcome these limitations, the assessment of VAC using a non-invasive, Doppler-derived, single-beat technique has been tested to estimate the aortic arch PWV (aa-PWV) [33]. This measurement requires a hand-drawn centreline in which errors may be produced [34,35]. The distribution of aa-PWV across the whole cardiovascular continuum, from healthy controls to patients with different HF phenotypes, has been recently provided, suggesting aa-PWV as a potential new tool for the stratification of patients at risk or with clinically manifest HF [32].

#### 1.1.4. Exercise Intolerance and Breath Analysis

Exercise intolerance, defined as the failure to perform physical activities without experiencing dyspnoea and/or fatigue, is a pivotal feature of cardiovascular abnormalities. Several factors, namely the cardiovascular system, the lungs, and peripheral muscles, must act in concert [36]. Cardiopulmonary exercise testing (CPET) is the gold standard for objectively investigating metabolic and ventilatory alterations responsible for exercise intolerance [37]. This technique is mainly applied to HF but may provide more accurate phenotyping of patients at different stages across the cardiovascular continuum [38]. Albeit exercise stress echocardiography (ESE) proved to have additional value in prognostic stratification of patients with various cardiovascular diseases [39,40], combining CPET-ESE can provide an even more integrated evaluation of metabolic and ventilatory responses to exercise with a more direct assessment of cardiovascular function. In the semi-supine position, the patient performs a symptom-limited graded ramp bicycle exercise test on a tilting microprocessor-controlled stress echocardiography cycle ergometer. The protocol comprises 2 min of unloaded [35] pedalling and 4 min of recovery after peak effort [41]. A 12-lead electrocardiogram and non-invasive arterial saturation are continuously monitored, and heart rate and brachial blood pressure are measured at rest and every minute during exercise. During the exercise, a comprehensive echocardiographic examination is performed concurrently with breath-by-breath gas exchange measurements, including speckle tracking and three-dimensional (3D) evaluation [15]. The assessments occur at rest and in three specific individual stages of effort: within the first 4 min of exercise (low load), after reaching a stable respiratory exchange ratio (RER ≥ 1.00), which is considered the anaerobic threshold (AT), and at peak effort [42,43]. Congestion, mainly using lung US, can be assessed during exercise. ΔB-lines have been included in a weighted score exercise-induced along with NT-proBNP, systolic pulmonary artery pressure and two indices derived from CPET (peak oxygen consumption [VO_2_] and ventilation [VE]/carbon dioxide production [VCO_2_] slope). The score improves the prognostic stratification of patients with cardiovascular risk at the risk of transition to overt HF, and those with a definite diagnosis of HF characterised by a poor outcome [44].

Finally, attention has been drawn to measuring additional volatile organic compounds during exercise. Indeed, a breath-by-breath analysis can be performed using a dedicated mass spectrometry instrument in addition to the traditional gas analyser of CPET. Patients with HF show increased breath levels of acetone, mirroring alterations of mitochondrial metabolism and oxidation of fatty acids [45]. The study of exhaled breath metabolites may represent a non-invasive method to improve our knowledge regarding metabolic derangement in CMDs [45].

### 1.2. Novel Molecular Targets

#### 1.2.1. Epigenetic Changes

Where environment represents the macroscopic link between ageing, metabolism, and CMDs, epigenetics is its molecular explanation. Briefly, it is the sum of regulatory mechanisms that can maintain alternative gene function/expression/activity without altering the DNA sequence. The epigenome, i.e., the complete profile of the individual epigenetic changes, is our personal diary of experiences, lifestyle changes, and encounters with diverse environmental influences. Epigenetic changes occur early and induce premature and persistent transcriptional changes that remain silent but contribute to the onset of CMDs since adolescence or even before [42,43].

Epigenetic changes might be divided into three major clusters: (i) DNA methylation, (ii) post-translational modifications of histone proteins, (iii) and noncoding RNA regulation [44].

#### 1.2.2. Long Noncoding RNAs

Over the past decade, various RNA sequencing experiments have revealed that many RNAs function as noncoding RNA (ncRNA) [46]: only 3% of the genome is represented by coding transcripts. Initially thought to be junk DNA [47,48] noncoding transcripts are now considered a class of functional molecules regulating cell differentiation, proliferation, apoptosis, necrosis, and autophagy [49] in every cell type involved in the development of CMDs: cardiomyocytes, endothelial cells (ECs), vascular smooth muscle cells, fibroblasts [46]. These emerging RNAs are classified into two classes according to their length: (i) small ncRNAs, longer than 200 nucleotides [including microRNAs (miRNA), transfer RNAs, and small nucleolar RNAs], and (ii) long ncRNAs (lcnRNAs), longer than 200 nucleotides [47]. Compared to miRNAs, lncRNAs are less abundant and less conserved. Still, their expression patterns are more cell type-specific and elicit specific activity due to their ability to control the localisation, interaction, and availability of effectors at a specific site [50]. Moreover, they are involved in several distinct roles: (i) inhibition/stimulation of gene expression by regulating mRNA/premRNA splicing; (ii) splicing in several miRNAs products; (iii) miRNA sponges (i.e., binding to a specific miRNA thus downregulating its function), (iv) co-expression of lncRNAs with miRNAs, (v) mutual repression of lncRNAs and miRNAs, (vi) the role of miRNAs as negative regulators of lncRNAs and (vii) protein–RNA interaction (e.g., post-translational modifications) [49]. These roles make them ideal candidates for regulating various pathophysiological and pathophysiological processes in CMDs [51,52].

LncRNA H19 prevents cardiomyocyte necrosis and hypertrophy [49,53]. Metastasis-associated lung adenocarcinoma transcript 1 (MALAT1) was upregulated in patients with myocardial infarction [54]. MALAT1 acts as an miRNA sponge, preserving mitochondria metabolism and function [54]. An extensive summary of the molecular mechanisms of major lncRNAs involved in CMDs can also be found in the works of Huang et al. and Statello et al. [49,52].

Regulating tissue- and disease-specific pathogenetic pathways, lncRNAs appear as ideal therapeutic targets. A recently identified lncRNA, named cardiomyocyte proliferation regulator, inhibits an initiator of eukaryotic genome replication and cell cycle progression. Cardiac-specific deletion of cardiomyocyte proliferation regulator increases cardiomyocyte proliferation, reduces myocardial injury-induced scar formation, and improves cardiac function in postnatal and adult hearts after myocardial injury [55].

What appears as a compelling avenue of research is the investigation of lncRNAs as circulating biomarkers in body fluids, informing on the disease several years before its clinical onset. Plasma levels of long intergenic noncoding RNA (LIPCAR) were associated with adverse outcomes in a case–control study (*n* = 198 patients; deceased vs. alive within 3 years from the blood draw). Higher levels (third and fourth quartiles) of LIPCAR were associated with increased cardiovascular mortality (OR, 6.58; 95% CI 2.76–16.67 and 13.23; 95% CI 5.19–36.8, respectively) [56]. In patients with acute myocardial infarction (*n* = 414), circulating levels of antisense noncoding RNA in the INK4 locus (ANRIL) and KCNQ1 opposite strand/antisense transcript 1 improved the prediction (integrated discrimination index +3%, +0.5% and +4% when considered together) of left ventricular dysfunction (ejection fraction 40%) onset in 4 months [57]. Thus, lncRNAs represent potent post-transcriptional modulators of gene expression with a high tissue specificity [58]. Therefore, identifying the specific lncRNAs involved in the heart and vessels (dys)function and assessing their circulating levels represents an important step towards precision in identifying patient-specific and disease-targeted therapies. Thus, lncRNAs represent potent post-transcriptional modulators of gene expression with a high tissue specificity [58].

#### 1.2.3. Sirtuins

Discovered for the first time in yeast, the silent mating type information regulation family (sirtuins) includes conserved proteins belonging to class III histone deacetylases with NAD+ as the required coenzyme [59]. Being involved in pathways regulating metabolism and mediating the beneficial effect of physical activity, healthy diet, and fasting-like conditions, several studies have extensively investigated their protective role in cardiometabolic diseases [60,61]. Among the different sirtuins, Sirt1 (silent mating type information regulation 1 homologs) has been most investigated in vivo and in humans (Table 2) [61]. Sirt1 regulates mitochondrial function [62], fatty acid metabolism [63], and inflammation [64], and is also involved in glucocorticoid signalling [65]. Sirt1 also suppresses senescence-associated secretory phenotype (SASP) transcription in cardiomyocytes. Conversely, a decrease in Sirt1 induces cell senescence and has been linked to the development of CMDs [66]: for instance, Sirt1 levels are reduced in the myocardium of individuals with metabolic cardiomyopathy [67]. In ECs, Sirt1 also maintains endothelial cell function by regulating endothelial nitric oxide synthase (eNOS) [60,61]. Targeting Sirt1 via exogenous activators or supplementation showed promising preclinical results. In a db/db mouse model, restoration of Sirt1 levels preserves systemic and metabolic cardiac function [67]. In line with this, the beneficial effects in cardiomyocytes of reducing myocardial hypertrophy and fibrosis by regulating NAD+ levels have been achieved by supplementation of alpha-ketoglutarate (AKG) [68], by genetic or resveratrol (RSV) activation of Sirt1 [69,70]. In the vasculature of the aged mice, restored Sirt1 activity via SRT1720 [71] and genetic activation or RSV [72] have shown beneficial effects on vascular disease and reverse vascular endothelial dysfunction, excessive superoxide production, inflammation, and arterial stiffness [73,74]. In human patients with obesity and no other comorbidities, SRT1720 rescues the ex vivo microvascular dysfunction via restoration of mitochondrial function, supporting the early targeting of Sirt1 pathways in the cardiometabolic disease [62]. Other clinical studies have shown that RSV promotes endothelial function, improves diastolic and systolic function, and reduces low-density lipoprotein levels and inflammatory biomarkers [75,76].

However, clinical trials in humans are still controversial. In patients with metabolic syndrome, RSV improves insulin sensitivity, reduces fat mass, and improves diastolic blood pressure [77]. In patients with T2D, it improves fasting blood sugar and decreases insulin resistance [78]. On the other hand, in glucose-intolerant patients, there is an improvement in vascular function, but there is no effect on insulin tolerance or sensitivity [79]. Similarly, SRT2104 treatment shows only mild positive effects when administered to patients with T2D, where it induces weight loss with no substantial improvement of endothelial function and lipid profiles [63,80].

**Table 2:** (**a**). Preclinical animal studies of Sirt1 in cardiometabolic disease. AKG: alpha-ketoglutarate; NFκB: nuclear factor kappa-light-chain-enhancer of activated B cells; rSirt1: recombinant silent mating type information regulation 1 homologs; RSV: resveratrol; Sirt1: silent mating type information regulation 1 homologs; ROS: reactive oxygen species; VCAM-1: vascular cell adhesion molecule-1. (**b**). Clinical human studies of Sirt1 in cardiometabolic disease. LDL: low-density lipoprotein; T2D: type 2 diabetes; VCAM-1: vascular cell adhesion molecule-1.

**(a)**				
**Drug**	**Model**	**Disease**	**Effects**	**References**
AKG	Pressure overload-induced mice	Heart failure	Increased mitophagy. Reduction of ferroptosis and cell damage.	[68]
rSirt1	db/db mouse	Metabolic cardiomyopathy	Restoration of Sirt1 levels. Improvement of left ventricular ejection fraction, fractional shortening, and diastolic function. Reduction of medium- and long-chain triacylglycerols containing saturated fatty acids. Increased triacylglycerols containing docosahexaenoic acid. Underregulated lipid trafficking and inflammation genes.	[67]
RSV	Diabetic mice + streptozotocin	Diabetic cardiomyopathy	Activation of Sirt1-dependent transcriptional regulatory mechanisms. Improvement of cardiac function.	[69]
	Accelerated vascular ageing rats	Vascular oxidative stress and inflammation	Protection against the harmful effect of ROS generation, proinflammatory mediators, and endothelial cell apoptosis. Sirt1 activation ameliorated endothelial stiffness by preventing p65-NFκB activation, VCAM-1 upregulation, and decreasing p47phox.	[72]
	Mice + high fat/high sucrose diet	Metabolic syndrome	Increased Sirt1 expression and activity, cyclooxygenase-2 expression, and antioxidant enzymes. Restoring vascular homeostasis and endothelial function. Reduction of NFκB acetyl-p65, vascular oxidative stress, and risk of cardiovascular disease.	[73]
SRT1720	Ageing mice	Vascular endothelial dysfunction.	Increased vascular relaxation and reduced superoxide and inflammation.	[74]
	Mice + high fat/high sucrose diet	Metabolic syndrome	Anti-inflammatory and antioxidant effects.	[73]
	Genetically obese mice	Metabolic syndrome	Increase lifespan and decrease serum glucose.	[61]
	Mice + high-fat diet	Metabolic syndrome	Reduced obesity, insulin resistance, hepatic steatosis. Increased longevity.	[61]
**(b)**				
**Drug**	**Study population**		**Effects**	**References**
SRT1720	Obese and ageing patients		Rescue of endothelial dysfunction by reduction p66^Shc^ expression and modulation of mitochondria respiratory chain.	[62]
RSV	Healthy individuals		Decreased expression of endothelial cells (intercellular adhesion molecule 1, VCAM-1 and interleukin 8) and inflammatory biomarkers (decreased level in plasma, interferon-gamma and insulin).	[75]
	Healthy obese men		Reduction in systolic blood pressure and increase in insulin sensitivity.	[61]
	Healthy non-obese men		No effects.	[61]
	Non-alcoholic fatty liver disease		Reduction in LDL, insulin resistance, hepatic steatosis and inflammation	[61]
	Metabolic syndrome patients		Reduction of fat mass. Improvement in diastolic blood pressure. Improvement of insulin sensitivity.	[77]
	T2D		Lowering of glucose levels. Decrease in insulin resistance.	[78]
	Patients with peripheral artery disease		No change in walking performance	[61]
SRT2104	T2D		Effects on cardiovascular measures are predominantly neutral. Weight reduction. Deterioration in glycaemic control.	[80]
	Healthy older volunteers		Reduction of serum LDL and triglycerides levels.	[61]
	Healthy smokers		Reduction of serum LDL and triglycerides levels.	[61]

Although preclinical evidence looks consistently promising, clinical interventions to restore/preserve sirtuins’ protective role are still far from being implemented. Considering the gender-related differences in the cardiovascular and longevity pathways, it is crucial to identify the ideal target (and the ideal timing) of intervention [61].

#### 1.2.4. Epigenetic Readers: Bromodomain and Extraterminal Domain (BET) Proteins

Epigenetic readers bind to acetylated histone tails and enable the coordination of the transcription machinery and specific transcriptional regulators, ultimately leading to the hyperexpression of the genes of interest [81]. BET proteins are a class of epigenetic readers involved in the hyperactivation of cancer [82,83] and inflammatory-related pathways [84]. Since inflammation is a crucial mediator of the initiation and progression of cardiometabolic damage, BET proteins, particularly bromodomain-containing protein 4 (BRD4), have been investigated as potential cardiovascular damage mediators [85]. BRD4 is involved in the endothelial cytokine storm during severe acute respiratory syndrome coronavirus 2 infection and the inflammatory response occurring in CMDs, affecting both cardiomyocytes and vascular endothelium [86,87]. In cardiomyocytes, BRD4 binding to acetylated RelA enhances activation of nuclear factor kappa-light-chain-enhancer of activated B cells (NFκB) signalling and BRD4 knockdown downregulates the natriuretic peptide A and natriuretic peptide B and reduce apoptosis [88,89]. In endothelial cells, it has been demonstrated the role of BRD4 as a coactivator of the NFκB RelA subunit, with the enrichment of RelA and BRD4 at the promoter and super-enhancer regions after proinflammatory stimuli [90]. Also, both in endothelial cells and in vivo in a streptozotocin mouse with an ischemic limb, BRD4 inhibition rescues physiologic angiogenesis [91]. Targeting BRD4 to blunt cardiovascular inflammation thus seems a promising strategy, and non-selective (pan-BET inhibitors) and selective (targeting BRD4 only) BET inhibitors (BETi) are being developed [92]. Duan et al. showed that JQ1 (a pan-BETi) ameliorates the cardiac profile of post-myocardial infarction HF mice via reduction of hypertrophic, inflammatory, and fibrotic responses [85]. However, it has also been reported that exposure of healthy male mice and rats to the BRD2-3-4 IBET-151 damaged cardiac mitochondria and led to dilated cardiomyopathy [93]. These contrasting results might imply (i) indirect cardiac effect of pharmacological intervention, (ii) off-target extracardiac effects, and (iii) discrepancy in the in vivo/in vitro models [94]. The first two points are particularly valid for pan-BETi, which still fails the clinical translation in humans due to toxicity [92]. Selective BRD4 inhibitor apabetalone (RVX-208) is the only BETi that has concluded a phase III clinical trial. Selectively targeting BRD4 in human patients, it attenuates vascular inflammatory response via repression of tumour necrosis factor-alpha (TNF-α), lipopolysaccharide or interleukin 1 beta pathways [87], but also affects systemically apolipoprotein A1 gene expression and increases high-density lipoproteins levels [95,96]. In a pooled analysis of phase II trials, RVX-208 reduced the relative risk (RR) of major adverse cardiac events (MACE) by 44% in patients with cardiovascular diseases, and this effect was higher (57%) in diabetic patients when added to a statin [91,92]. In the phase III trial BETonMACE, a non-significant (*p* = 0.11) positive trend (18% RR) MACE reduction was seen in 2425 patients [97]. However, its capacity to blunt endothelial inflammation led to an FDA breakthrough approval for coronavirus 2 infection after major work pointing out RVX-208 as an ideal candidate to stop the cytokine storm [98]. All these findings suggest that BRD4 inhibition, especially at the vascular level, might be a key strategy to halt the progression of CMDs. Still, the conflicting and unclear evidence demands further in-depth investigation.

#### 1.2.5. Cellular Senescence

Senescence is a cellular response defined by cell cycle arrest associated with phenotypic alterations, including a proinflammatory secretome. Senescence plays a role in normal development, maintains tissue homeostasis, and limits tumour progression [99]. Nevertheless, premature senescence has a tight relationship with cardiometabolic damage [100]. In the senescence transition, cardiomyocytes and endothelial cells show common damage traits. Major phenotypic changes involved in the senescence shift and related to CMDs are (i) glycolytic shift [11], (ii) secretion of senescence-associated factors [100,101], and (iii) telomere shortening [102]. A comprehensive description of the latter can be found in these recent publications [103,104]. The metabolic remodelling of cardiomyocytes due to the shift from oxidative phosphorylation to glycolysis is associated with mitochondrial dysfunction and contributes to impaired cardiac contraction. It promotes the development of cardiomyopathies or arrhythmia [105]. In particular, activation of glycolysis follows activation of p300/CREB binding protein (CBP), a histone acyltransferase that upregulates the enhancer region of *Hexokinase 2* [106], which in turn contributes to the recruitment of BRD4 to nucleosomes flanking transcription factor binding sites. Cellular senescence is also characterised by the secretion of secretory phenotypes associated with SASP as soluble signalling factors, proinflammatory cytokines, chemokines, extracellular matrix-degrading proteins, and others. These cause chronic inflammation and tissue destruction [100,107]. Although extensively studied in other cell types (e.g., inflammatory cells), there is limited evidence for the role of SASP in cardiomyocytes. For instance, senescence and SASP were identified as the main mechanisms involved in the cardiac degenerative defect in diabetes, and they contributed to a deficit in the regenerative potential and ability of cardiac stem cells to differentiate into new cardiomyocytes [108].

Evidence shows that aged cardiomyocytes increase SASP factors that regulate the local non-myocyte microenvironment and contribute to cardiac remodelling and dysfunction [105]. The non-myocyte population includes endothelial cells, which secrete endothelium-specific paracrine, angiocrine, and proinflammatory factors. With exosome production, endothelial cells modulate cardiomyocyte function, maturation, haemostasis, and senescence [105].

Pharmacological targeting of senescence has rapidly grown over the last few years, even though limited evidence is available for CMDs [109]. Senotherapeutic drugs fall into two categories: (i) senolytics, named for their ability to lysis of senescent cells, and (ii) senomorphs, also known as gerosuppressors, which inhibit SASP secretion [110]. Senolytics are compounds that can promote the apoptosis of senescent cells in vitro and affect the reduction of senescent cells in vivo. Noteworthy, established drugs that show senolytics effects are statins, which are widely used in patients at high cardiovascular risk. Statins exert a major cytotoxic effect on senescent rather than non-senescent human vascular cells via modulation of the mevalonate pathway [111]. Eliminating senescent endothelial cells is a beneficial effect [101], and directly targeting senescent cells (i.e., with senescence-specific senolytics) [101,112] might be the ideal approach. Belcastro and colleagues developed theranostic nanocarriers (NC) decorated with VCAM-1 antibodies (NC-VCAM-1) to target cell surface VCAM-1, which is overexpressed in senescent ECs [112].

Although no senomorphic drugs specifically targeting cardiac endothelial cells and cardiomyocytes have been identified, their beneficial effects in treating cellular senescence in other contexts have been extensively investigated. Relevant examples include rapamycin or its analogues, which have been shown to prevent senescence in vivo and attenuate SASP [113]. Metformin, a cardiometabolic-specific drug, also deserves mention since it improves Sirt6 levels in pericoronary fat [114,115]. Sirt6 levels are also ameliorated in carotid plaques by sodium–glucose cotransporter 2 inhibitors, an emerging class of antidiabetic drugs [116].

In conclusion, although still relatively uncharted territory in the cardiometabolic context, senescence could represent both a source of novel biomarkers and new molecular targets. Addressing the tissue senescence rather than just the tissue candidates has become a novel concept in cardiometabolic-related disease treatment.

## 2. Conclusions: Gap of Knowledge and Next Steps

Molecular biomarkers and metabolic indices could help elucidate the multifaceted mechanisms associated with many cardiometabolic diseases (CMDs) [37,117] by adopting a feasible approach that identifies patients in a sub-clinical condition with a robust prognostic value [19].

Evaluating congestion through ultrasound (US), along with epicardial adipose tissue (EAT) assessment and ventricular–arterial coupling (VAC) markers in a multimodal approach, may complete the real-time evaluation of patients with CMDs [22]. Further studies are needed to implement additional US measurements of congestion, e.g., jugular, portal, and hepatic vein US [22], together with other biomarkers such as atrial natriuretic peptide (ANP), high-sensitivity cardiac troponin or soluble ST2 [26]. Cardiopulmonary exercise testing (CPET) now has a definitive place in the armamentarium of the practising clinician for the evaluation of cardiopulmonary disorders, primarily heart failure (HF). It provides a thorough assessment of the integrative multiorgan physiological response to exercise. A revival of invasive CPET and the introduction of CPET imaging have extended the amount of pathophysiological and clinical information, providing new insights into systemic and pulmonary hemodynamics and direct knowledge of cardiac, valve, and functional data [118].

**Figure 2:**
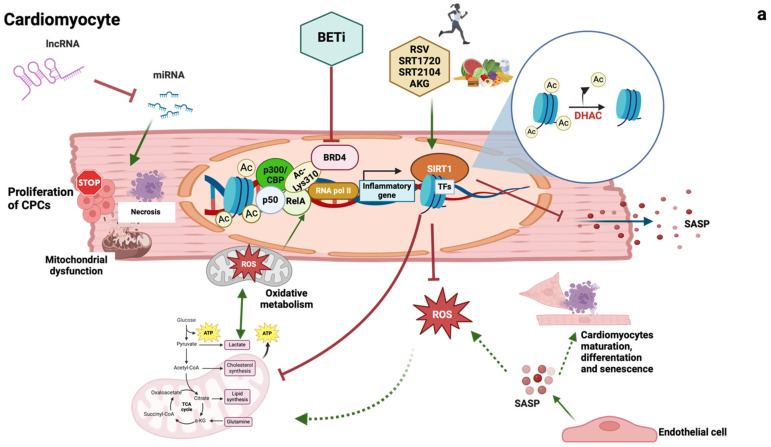

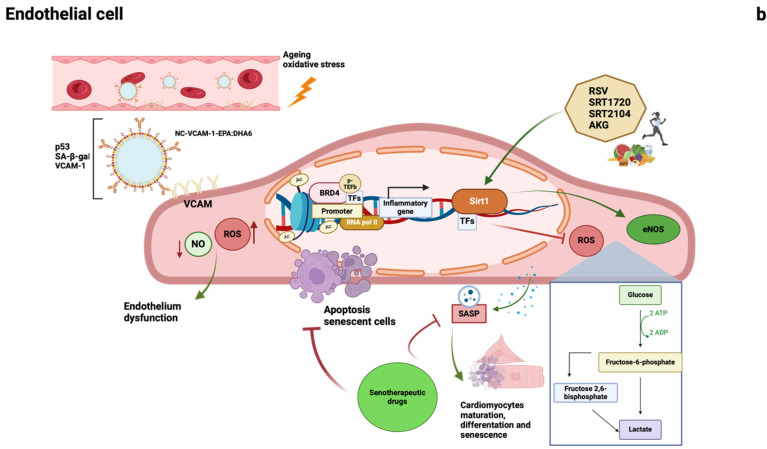
Major molecular targets in cardiomyocytes (**a**) and endothelial cells (**b**). By binding the corresponding microRNA (miRNA) and inhibiting this, the long noncoding RNA (lncrna) acts as a miRNA sponge and could prevent cardiomyocyte necrosis and hypertrophic growth, rescuing cardiac progenitor cell (CPC) profiling and preventing mitochondrial dysfunction. Bromodomain-containing protein 4 (BRD4) binds to RelA by recognising the acetylene-lysine residue and initiates the transcription of genes involved in the inflammatory process. The shift from oxidative phosphorylation to glycolysis is associated with mitochondrial dysfunction. In addition, the activation of glycolysis follows the activation of p300/CREB binding protein (CBP), which contributes to the recruitment of BRD4 to nucleosomes flanking transcription factor (TF) binding sites. The effect of BRD4 could be prevented by BETi inhibitors (BETi). Silent mating type information regulation 1 homologs (Sirt1) can repress the transcription of a senescence-associated secretory phenotype (SASP) through histone deacetylation. Sirt1 activity can be stimulated by an active lifestyle, healthy diet, and compounds such as resveratrol (RSV), SRT1720, SRT2104, and alpha-ketoglutarate (AKG) (**a**,**b**). Reactive oxygen species (ROS) production, in turn, contributes to the metabolic shift towards oxidative metabolism (**a**). Excess glycolysis associated with endothelial dysfunction could be prevented by activating Sirt1. Sirt1 regulates endothelial nitric oxide synthase (eNOS) and endothelial function. Theranostic nanocarriers (NCs) loaded with omega-3 polyunsaturated fatty acids [NC–EPA:DHA (6:1)]. NC prevents VCAM-1, upregulation of p53, senescence-associated β-galactosidase activity (SA-β-galactosidase). SASP from endothelial cells and exosome production modulate cardiomyocyte function, maturation, haemostasis, and senescence (**a**,**b**), and senotherapeutic drugs act by inhibiting the secretion of SASP or promoting apoptosis of senescent cells. In endothelial cells, Sirt-1 maintains endothelial cell function by regulating endothelial eNOS. P-TEFb: transcription elongation factor; NO: nitric oxide.

In recent years, numerous basic research studies have identified novel pathways and specific targets related to cardiometabolic diseases (Figure 2). However, their translation into clinical practice is not imminent due to a significant knowledge gap regarding their true significance and mechanisms in the human species. Epigenetics, and in particular long non-coding RNAs (lncRNAs) and chromatin modulation (e.g., bromodomain and extra-terminal inhibitors (BETi) and sirtuins), may be close to filling this gap, as evidenced by several epigenetic drugs in development that have already been successfully tested in vivo and even in phase III trials [50]. On the other hand, senescence seems to be the least explored but the most fascinating, given the potential to target not a tissue but only its senescent part. Several points are still missing, as demonstrated by the difficulties in defining senescence in cardiomyocytes, but there is evidence that senescence may be a major contributor to cardiovascular disease [119]. This has highlighted the need to elucidate aspects of senescence biology and its role in the cardiac cell population to take advantage of the senolytic drugs already in use [119].

In conclusion, while the road ahead seems challenging and complex, it is also filled with promise, making the prospect of precision medicine increasingly attainable. The current advances in cardiology together with the unresolved problems that the new biomarkers may help to understand, are leading to a more personalised and effective treatment of CMDs.

## Figures and Tables

**Table 1:** **Novel techniques and biomarkers to evaluate cardiometabolic diseases in the clinical setting.** Abbreviations: 2D: 2-dimensional; aa-PWV: aortic arch pulse wave velocity; ANP: atrial natriuretic peptide; cf-PWV: carotid–femoral pulse wave velocity; CPET: cardiopulmonary exercise testing; ESE: exercise stress echocardiography; HFpEF: heart failure with preserved ejection fraction; HFrEF: heart failure with reduced ejection fraction; hs-Tn: high-sensitivity troponin; LUS: lung ultrasound; MCP-1: monocyte chemoattractant protein-1; sST2: suppression of tumorigenicity 2 protein; TNF-α: tumour necrosis factor α.

Main Group	Traditional Biomarkers	Novel Biomarkers	Value and New Perspectives
Epicardial adiposity	2D echocardiography Magnetic resonance Computed tomography	TNF-α Resistin MCP-1 Adiponectin	Characterising the inflammatory-metabolic phenotype of HFpEF It is associated with atrial and ventricular fibrosis Estimating cardiac cachexia in patients with HFrEF
Congestion	Natriuretic peptide: Ultrasound: Inferior vena cava Jugular vein distention Renal venous flow LUS	ANP hs-Tn sST2	Predicting the trajectory of subclinical damage like cardiac remodelling
Ventricular–arterial coupling	Arterial elastance/LV elastance (cardiac catheterisation) cf-PWV (tonometry)	aa-PWV Aortic characteristic impedance Aortic distensibility Beta stiffness index	Phenotyping hypertensive patients with reduced exercise capacity Predicting mortality following hospitalisation among HFpEF patients
Exercise capacity	CPET	CPET-ESE (+LUS) Breath analysis of fatty acids oxidation and oxidative stress metabolites	Investigating the pathophysiologic mechanisms underpinning exercise intolerance Improving risk stratification

## Abbreviations

AKG	alpha-ketoglutarate
ANRIL	antisense non-coding RNA in the INK4 Locus
BET	bromodomain and extraterminal proteins BETi inhibitors (BETi)
BRD4	bromodomain-containing protein 4
CBP	CREB-binding protein
LIPCAR	long intergenic noncoding RNA predicting CARdiac remodelling
lncRNA	long noncoding RNA
MALAT 1	metastasis-associated lung adenocarcinoma transcript 1
MCM	metabolic cardiomyopathy
ncRNA	noncoding RNA
NAD	nicotinamide adenine dinucleotide
NC	nano carrier
NFκB	nuclear factor kappa-light-chain-enhancer of activated B cells
rSirt1	recombinant Sirt1
RSV	resveratrol
RVX-208	apabetalone
SASP	senescent-associated secretory phenotype
Sirt1	silent mating type information regulation 1 homologs

## References

[1] Di Angelantonio E., Kaptoge S., Wormser D., Willeit P., Butterworth A.S., Bansal N., O’Keeffe L.M., Gao P., Wood A.M., Burgess S. (2015). Association of cardiometabolic multimorbidity with mortality. JAMA—J. Am. Med. Assoc..

[2] Cheng X., Ma T., Ouyang F., Zhang G., Bai Y. (2022). Trends in the Prevalence of Cardiometabolic Multimorbidity in the United States, 1999–2018. Int. J. Environ. Res. Public Health.

[3] Oishi Y., Manabe I. (2020). Organ System Crosstalk in Cardiometabolic Disease in the Age of Multimorbidity. Front. Cardiovasc. Med..

[4] Pallarés-Carratalá V., Ruiz-García A., Serrano-Cumplido A., Arranz-Martínez E., Divisón-Garrote J.A., Moyá-Amengual A., Escobar-Cervantes C., Barrios V. (2023). Prevalence Rates of Arterial Hypertension According to the Threshold Criteria of 140/90 or 130/80 mmHg and Associated Cardiometabolic and Renal Factors: SIMETAP-HTN Study. Medicina.

[5] Redon J., Cifkova R., Laurent S., Nilsson P., Narkiewicz K., Erdine S., Mancia G. (2009). Mechanisms of hypertension in the cardiometabolic syndrome. J. Hypertens..

[6] Balletti A., De Biase N., Del Punta L., Filidei F., Armenia S., Masi F., Di Fiore V., Mazzola M., Bacca A., Dini F.L. (2023). Cardiometabolic Phenotyping in Heart Failure: Differences between Patients with Reduced vs. Preserved Ejection Fraction. Diagnostics.

[7] Schwinger R.H.G. (2021). Pathophysiology of heart failure. Cardiovasc. Diagn. Ther..

[8] McDonagh T.A., Metra M., Adamo M., Gardner R.S., Baumbach A., Böhm M., Burri H., Butler J., Celutkiene J., Chioncel O. (2021). 2021 ESC Guidelines for the diagnosis and treatment of acute and chronic heart failure. Eur. Heart J..

[9] Serio S., Pagiatakis C., Musolino E., Felicetta A., Carullo P., Laura Frances J., Papa L., Rozzi G., Salvarani N., Miragoli M. (2023). Cardiac Aging Is Promoted by Pseudohypoxia Increasing p300-Induced Glycolysis. Circ. Res..

[10] Ponikowski P., Voors A.A., Anker S.D., Bueno H., Cleland J.G.F., Coats A.J.S., Falk V., González-Juanatey J.R., Harjola V.-P., Jankowska E.A. (2016). 2016 ESC Guidelines for the diagnosis and treatment of acute and chronic heart failure. Eur. Heart J..

[11] Iacobellis G. (2022). Epicardial adipose tissue in contemporary cardiology. Nat. Rev. Cardiol..

[12] Iacobellis G., Willens H.J. (2009). Echocardiographic Epicardial Fat: A Review of Research and Clinical Applications. J. Am. Soc. Echocardiogr..

[13] Packer M. (2018). Epicardial Adipose Tissue May Mediate Deleterious Effects of Obesity and Inflammation on the Myocardium. J. Am. Coll. Cardiol..

[14] Bozkurt B., Coats A.J.S., Tsutsui H., Abdelhamid C.M., Adamopoulos S., Albert N., Anker S.D., Atherton J., Böhm M., Butler J. (2021). Universal definition and classification of heart failure: A report of the Heart Failure Society of America, Heart Failure Association of the European Society of Cardiology, Japanese Heart Failure Society and Writing Committee of the Universal Definition of heart failure. Eur. J. Heart Fail..

[15] Pugliese N.R., Paneni F., Mazzola M., De Biase N., Del Punta L., Gargani L., Mengozzi A., Virdis A., Nesti L., Taddei S. (2021). Impact of epicardial adipose tissue on cardiovascular haemodynamics, metabolic profile, and prognosis in heart failure. Eur. J. Heart Fail..

[16] Gorter T.M., van Woerden G., Rienstra M., Dickinson M.G., Hummel Y.M., Voors A.A., Hoendermis E.S., van Veldhuisen D.J. (2020). Epicardial Adipose Tissue and Invasive Hemodynamics in Heart Failure with Preserved Ejection Fraction. JACC Heart Fail..

[17] Verbrugge F.H., Omote K., Reddy Y.N.V., Sorimachi H., Obokata M., Borlaug B.A. (2022). Heart failure with preserved ejection fraction in patients with normal natriuretic peptide levels is associated with increased morbidity and mortality. Eur. Heart J..

[18] Núñez J., de la Espriella R., Rossignol P., Voors A.A., Mullens W., Metra M., Chioncel O., Januzzi J.L., Mueller C., Richards A.M. (2022). Congestion in heart failure: A circulating biomarker-based perspective. A review from the Biomarkers Working Group of the Heart Failure Association, European Society of Cardiology. Eur. J. Heart Fail..

[19] Mullens W., Damman K., Harjola V.P., Mebazaa A., Brunner-La Rocca H.P., Martens P., Testani J.M., Tang W.H.H.W., Orso F., Rossignol P. (2019). The use of diuretics in heart failure with congestion—A position statement from the Heart Failure Association of the European Society of Cardiology. Eur. J. Heart Fail..

[20] Felker G.M., Anstrom K.J., Adams K.F., Ezekowitz J.A., Fiuzat M., Houston-Miller N., Januzzi J.L., Mark D.B., Piña I.L., Passmore G. (2017). Effect of natriuretic peptide–guided therapy on hospitalization or cardiovascular mortality in high-risk patients with heart failure and reduced ejection fraction: A randomized clinical trial. JAMA—J. Am. Med. Assoc..

[21] Tsutsui H., Albert N.M., Coats A.J.S., Anker S.D., Bayes-Genis A., Butler J., Chioncel O., Defilippi C.R., Drazner M.H., Felker G.M. (2023). Natriuretic peptides: Role in the diagnosis and management of heart failure: A scientific statement from the Heart Failure Association of the European Society of Cardiology, Heart Failure Society of America and Japanese Heart Failure Society. Eur. J. Heart Fail..

[22] Pugliese N.R., Pellicori P., Filidei F., Del Punta L., De Biase N., Balletti A., Di Fiore V., Mengozzi A., Taddei S., Gargani L. (2023). The incremental value of multi-organ assessment of congestion using ultrasound in outpatients with heart failure. Eur. Heart J.—Cardiovasc. Imaging.

[23] Correale M., Fioretti F., Tricarico L., Croella F., Brunetti N.D., Inciardi R.M., Mattioli A.V., Nodari S. (2023). The Role of Congestion Biomarkers in Heart Failure with Reduced Ejection Fraction. J. Clin. Med..

[24] Chubuchny V., Pugliese N.R., Taddei C., Poggianti E., Spini V., Barison A., Formichi B., Airò E., Bauleo C., Prediletto R. (2021). A novel echocardiographic method for estimation of pulmonary artery wedge pressure and pulmonary vascular resistance. ESC Heart Fail..

[25] Pellicori P., Platz E., Dauw J., ter Maaten J.M., Martens P., Pivetta E., Cleland J.G.F., McMurray J.J.V., Mullens W., Solomon S.D. (2021). Ultrasound imaging of congestion in heart failure: Examinations beyond the heart. Eur. J. Heart Fail..

[26] Murphy S.P., Prescott M.F., Camacho A., Iyer S.R., Maisel A.S., Felker G.M., Butler J., Piña I.L., Ibrahim N.E., Abbas C. (2021). Atrial Natriuretic Peptide and Treatment with Sacubitril/Valsartan in Heart Failure with Reduced Ejection Fraction. JACC Heart Fail..

[27] Ikonomidis I., Aboyans V., Blacher J., Brodmann M., Brutsaert D.L., Chirinos J.A., De Carlo M., Delgado V., Lancellotti P., Lekakis J. (2019). The role of ventricular–arterial coupling in cardiac disease and heart failure: Assessment, clinical implications and therapeutic interventions. A consensus document of the European Society of Cardiology Working Group on Aorta & Peripheral Vascular Diseas. Eur. J. Heart Fail..

[28] Borlaug B.A., Melenovsky V., Redfield M.M., Kessler K., Chang H.J., Abraham T.P., Kass D.A. (2007). Impact of Arterial Load and Loading Sequence on Left Ventricular Tissue Velocities in Humans. J. Am. Coll. Cardiol..

[29] Ikonomidis I., Tzortzis S., Triantafyllidi H., Parissis J., Papadopoulos C., Venetsanou K., Trivilou P., Paraskevaidis I., Lekakis J. (2015). Association of impaired left ventricular twisting-untwisting with vascular dysfunction, neurohumoral activation and impaired exercise capacity in hypertensive heart disease. Eur. J. Heart Fail..

[30] Vinereanu D., Nicolaides E., Boden L., Payne N., Jones C.J.H., Fraser A.G. (2003). Conduit arterial stiffness is associated with impaired left ventricular subendocardial function. Heart.

[31] Shah A.M., Claggett B., Sweitzer N.K., Shah S.J., Anand I.S., Liu L., Pitt B., Pfeffer M.A., Solomon S.D. (2015). Prognostic importance of impaired systolic function in heart failure with preserved ejection fraction and the impact of spironolactone. Circulation.

[32] Pugliese N.R., Balletti A., Armenia S., De Biase N., Faita F., Mengozzi A., Paneni F., Ruschitzka F., Virdis A., Ghiadoni L. (2022). Ventricular-Arterial Coupling Derived from Proximal Aortic Stiffness and Aerobic Capacity across the Heart Failure Spectrum. JACC Cardiovasc. Imaging.

[33] Lang R.M., Badano L.P., Mor-Avi V., Afilalo J., Armstrong A., Ernande L., Flachskampf F.A., Foster E., Goldstein S.A., Kuznetsova T. (2015). Recommendations for cardiac chamber quantification by echocardiography in adults: An update from the American Society of Echocardiography and the European Association of Cardiovascular Imaging. Eur. Heart J. Cardiovasc. Imaging.

[34] Del Punta L., De Biase N., Balletti A., Filidei F., Pieroni A., Armenia S., Mengozzi A., Mazzola M., Di Fiore V., Dini F.L. (2022). Arterial Hypertension and Cardiopulmonary Function: The Value of a Combined Cardiopulmonary and Echocardiography Stress Test. High Blood Press. Cardiovasc. Prev..

[35] Pugliese N.R., Mazzola M., Fabiani I., Gargani L., De Biase N., Pedrinelli R., Natali A., Dini F.L. (2020). Haemodynamic and metabolic phenotyping of hypertensive patients with and without heart failure by combining cardiopulmonary and echocardiographic stress test. Eur. J. Heart Fail..

[36] Del Buono M.G., Arena R., Borlaug B.A., Carbone S., Canada J.M., Kirkman D.L., Garten R., Rodriguez-Miguelez P., Guazzi M., Lavie C.J. (2019). Exercise Intolerance in Patients with Heart Failure: JACC State-of-the-Art Review. J. Am. Coll. Cardiol..

[37] Del Punta L., De Biase N., Di Fiore V., Maremmani D., Gargani L., Mazzola M., De Carlo M., Mengozzi A., Lomonaco T., Galeotti G.G. (2023). Combining cardiopulmonary exercise testing with echocardiography: A multiparametric approach to the cardiovascular and cardiopulmonary systems. Eur. Heart J.—Imaging Methods Pract..

[38] Guazzi M., Myers J., Arena R. (2005). Cardiopulmonary exercise testing in the clinical and prognostic assessment of diastolic heart failure. J. Am. Coll. Cardiol..

[39] Gargani L., Pugliese N.R., De Biase N., Mazzola M., Agoston G., Arcopinto M., Argiento P., Armstrong W.F., Bandera F., Cademartiri F. (2023). Exercise Stress Echocardiography of the Right Ventricle and Pulmonary Circulation. J. Am. Coll. Cardiol..

[40] Fabiani I., Pugliese N.R., Galeotti G.G., D’Agostino A., Mazzola M., Pedrinelli R., Dini F.L. (2019). The Added Value of Exercise Stress Echocardiography in Patients with Heart Failure. Am. J. Cardiol..

[41] Nesti L., Pugliese N.R., Santoni L., Armenia S., Chiriacò M., Sacchetta L., De Biase N., Del Punta L., Masi S., Tricò D. (2024). Distinct effects of type 2 diabetes and obesity on cardiopulmonary performance. Diabetes Obes. Metab..

[42] Costantino S., Mohammed S.A., Ambrosini S., Paneni F. (2019). Epigenetic processing in cardiometabolic disease. Atherosclerosis.

[43] Švorcová J. (2023). Transgenerational Epigenetic Inheritance of Traumatic Experience in Mammals. Genes.

[44] Shi Y., Zhang H., Huang S., Yin L., Wang F., Luo P., Huang H. (2022). Epigenetic regulation in cardiovascular disease: Mechanisms and advances in clinical trials. Signal Transduct. Target. Ther..

[45] Biagini D., Pugliese N.R., Vivaldi F.M., Ghimenti S., Lenzi A., De Angelis F., Ripszam M., Bruderer T., Armenia S., Cappeli F. (2023). Breath analysis combined with cardiopulmonary exercise testing and echocardiography for monitoring heart failure patients: The AEOLUS protocol. J. Breath Res..

[46] Anderson K.M., Anderson D.M. (2022). LncRNAs at the heart of development and disease. Mamm. Genome.

[47] Uchida S., Dimmeler S. (2015). Long noncoding RNAs in cardiovascular diseases. Circ. Res..

[48] Struhl K. (2007). Transcriptional noise and the fidelity of initiation by RNA polymerase II. Nat. Struct. Mol. Biol..

[49] Huang Y. (2018). The novel regulatory role of lncRNA-miRNA-mRNA axis in cardiovascular diseases. J. Cell. Mol. Med..

[50] Lozano-Vidal N., Bink D.I., Boon R.A. (2019). Long noncoding RNA in cardiac aging and disease. J. Mol. Cell Biol..

[51] Rech M., Barandiarán Aizpurua A., van Empel V., van Bilsen M., Schroen B. (2018). Pathophysiological understanding of HFpEF: MicroRNAs as part of the puzzle. Cardiovasc. Res..

[52] Statello L., Guo C.-J., Chen L.-L., Huarte M. (2021). Gene regulation by long non-coding RNAs and its biological functions. Nat. Rev. Mol. Cell Biol..

[53] Liu L., An X., Li Z., Song Y., Li L., Zuo S., Liu N., Yang G., Wang H., Cheng X. (2016). The H19 long noncoding RNA is a novel negative regulator of cardiomyocyte hypertrophy. Cardiovasc. Res..

[54] Vausort M., Wagner D.R., Devaux Y. (2014). Long noncoding RNAs in patients with acute myocardial infarction. Circ. Res..

[55] Ponnusamy M., Liu F., Zhang Y.-H., Li R.-B., Zhai M., Liu F., Zhou L.-Y., Liu C.-Y., Yan K.-W., Dong Y.-H. (2019). Long Noncoding RNA CPR (Cardiomyocyte Proliferation Regulator) Regulates Cardiomyocyte Proliferation and Cardiac Repair. Circulation.

[56] Kumarswamy R., Bauters C., Volkmann I., Maury F., Fetisch J., Holzmann A., Lemesle G., de Groote P., Pinet F., Thum T. (2014). Circulating long noncoding RNA, LIPCAR, predicts survival in patients with heart failure. Circ. Res..

[57] Beermann J., Piccoli M.-T., Viereck J., Thum T. (2016). Non-coding RNAs in Development and Disease: Background, Mechanisms, and Therapeutic Approaches. Physiol. Rev..

[58] Ounzain S., Micheletti R., Beckmann T., Schroen B., Alexanian M., Pezzuto I., Crippa S., Nemir M., Sarre A., Johnson R. (2015). Genome-wide profiling of the cardiac transcriptome after myocardial infarction identifies novel heart-specific long non-coding RNAs. Eur. Heart J..

[59] Wu Q.-J., Zhang T.-N., Chen H.-H., Yu X.-F., Lv J.-L., Liu Y.-Y., Liu Y.-S., Zheng G., Zhao J.-Q., Wei Y.-F. (2022). The sirtuin family in health and disease. Signal Transduct. Target. Ther..

[60] Conti V., Corbi G., Polito M.V., Ciccarelli M., Manzo V., Torsiello M., De Bellis E., D’Auria F., Vitulano G., Piscione F. (2020). Sirt1 Activity in PBMCs as a Biomarker of Different Heart Failure Phenotypes. Biomolecules.

[61] Kane A.E., Sinclair D.A. (2018). Sirtuins and NAD(+) in the Development and Treatment of Metabolic and Cardiovascular Diseases. Circ. Res..

[62] Mengozzi A., Costantino S., Paneni F., Duranti E., Nannipieri M., Mancini R., Lai M., La Rocca V., Puxeddu I., Antonioli L. (2022). Targeting SIRT1 Rescues Age- and Obesity-Induced Microvascular Dysfunction in Ex Vivo Human Vessels. Circ. Res..

[63] Yamamoto T., Sano M. (2022). Deranged Myocardial Fatty Acid Metabolism in Heart Failure. Int. J. Mol. Sci..

[64] Yang Y., Liu Y., Wang Y., Chao Y., Zhang J., Jia Y., Tie J., Hu D. (2022). Regulation of SIRT1 and Its Roles in Inflammation. Front. Immunol..

[65] Sung J.Y., Kim S.G., Kim J.-R., Choi H.C. (2020). Prednisolone suppresses adriamycin-induced vascular smooth muscle cell senescence and inflammatory response via the SIRT1-AMPK signaling pathway. PLoS ONE.

[66] Jęśko H., Wencel P., Strosznajder R.P., Strosznajder J.B. (2017). Sirtuins and Their Roles in Brain Aging and Neurodegenerative Disorders. Neurochem. Res..

[67] Costantino S., Mengozzi A., Velagapudi S., Mohammed S.A., Gorica E., Akhmedov A., Mongelli A., Pugliese N.R., Masi S., Virdis A. (2023). Treatment with recombinant Sirt1 rewires the cardiac lipidome and rescues diabetes-related metabolic cardiomyopathy. Cardiovasc. Diabetol..

[68] Yu H., Gan D., Luo Z., Yang Q., An D., Zhang H., Hu Y., Ma Z., Zeng Q., Xu D. (2024). α-Ketoglutarate improves cardiac insufficiency through NAD(+)-SIRT1 signaling-mediated mitophagy and ferroptosis in pressure overload-induced mice. Mol. Med..

[69] Sulaiman M., Matta M.J., Sunderesan N.R., Gupta M.P., Periasamy M., Gupta M. (2010). Resveratrol, an activator of SIRT1, upregulates sarcoplasmic calcium ATPase and improves cardiac function in diabetic cardiomyopathy. Am. J. Physiol. Heart Circ. Physiol..

[70] Prola A., Pires Da Silva J., Guilbert A., Lecru L., Piquereau J., Ribeiro M., Mateo P., Gressette M., Fortin D., Boursier C. (2017). SIRT1 protects the heart from ER stress-induced cell death through eIF2α deacetylation. Cell Death Differ..

[71] Nandave M., Acharjee R., Bhaduri K., Upadhyay J., Rupanagunta G.P., Ansari M.N. (2023). A pharmacological review on SIRT 1 and SIRT 2 proteins, activators, and inhibitors: Call for further research. Int. J. Biol. Macromol..

[72] Csiszar A., Labinskyy N., Podlutsky A., Kaminski P.M., Wolin M.S., Zhang C., Mukhopadhyay P., Pacher P., Hu F., de Cabo R. (2008). Vasoprotective effects of resveratrol and SIRT1: Attenuation of cigarette smoke-induced oxidative stress and proinflammatory phenotypic alterations. Am. J. Physiol. Heart Circ. Physiol..

[73] Fry J.L., Al Sayah L., Weisbrod R.M., Van Roy I., Weng X., Cohen R.A., Bachschmid M.M., Seta F. (2016). Vascular Smooth Muscle Sirtuin-1 Protects against Diet-Induced Aortic Stiffness. Hypertension.

[74] Gano L.B., Donato A.J., Pasha H.M., Hearon C.M.J., Sindler A.L., Seals D.R. (2014). The SIRT1 activator SRT1720 reverses vascular endothelial dysfunction, excessive superoxide production, and inflammation with aging in mice. Am. J. Physiol. Heart Circ. Physiol..

[75] Agarwal B., Campen M.J., Channell M.M., Wherry S.J., Varamini B., Davis J.G., Baur J.A., Smoliga J.M. (2013). Resveratrol for primary prevention of atherosclerosis: Clinical trial evidence for improved gene expression in vascular endothelium. Int. J. Cardiol..

[76] Bonkowski M.S., Sinclair D.A. (2016). Slowing ageing by design: The rise of NAD(+) and sirtuin-activating compounds. Nat. Rev. Mol. Cell Biol..

[77] Méndez-del Villar M., González-Ortiz M., Martínez-Abundis E., Pérez-Rubio K.G., Lizárraga-Valdez R. (2014). Effect of resveratrol administration on metabolic syndrome, insulin sensitivity, and insulin secretion. Metab. Syndr. Relat. Disord..

[78] Movahed A., Nabipour I., Lieben Louis X., Thandapilly S.J., Yu L., Kalantarhormozi M., Rekabpour S.J., Netticadan T. (2013). Antihyperglycemic effects of short term resveratrol supplementation in type 2 diabetic patients. Evid.-Based Complement. Altern. Med..

[79] Pollack R.M., Barzilai N., Anghel V., Kulkarni A.S., Golden A., O’Broin P., Sinclair D.A., Bonkowski M.S., Coleville A.J., Powell D. (2017). Resveratrol Improves Vascular Function and Mitochondrial Number but Not Glucose Metabolism in Older Adults. J. Gerontol. A Biol. Sci. Med. Sci..

[80] Noh R.M., Venkatasubramanian S., Daga S., Langrish J., Mills N.L., Lang N.N., Hoffmann E., Waterhouse B., Newby D.E., Frier B.M. (2017). Cardiometabolic effects of a novel SIRT1 activator, SRT2104, in people with type 2 diabetes mellitus. Open Heart.

[81] Shvedunova M., Akhtar A. (2022). Modulation of cellular processes by histone and non-histone protein acetylation. Nat. Rev. Mol. Cell Biol..

[82] Xu Y., Vakoc C.R. (2017). Targeting Cancer Cells with BET Bromodomain Inhibitors. Cold Spring Harb. Perspect. Med..

[83] Shu S., Lin C.Y., He H.H., Witwicki R.M., Tabassum D.P., Roberts J.M., Janiszewska M., Huh S.J., Liang Y., Ryan J. (2016). Response and resistance to BET bromodomain inhibitors in triple-negative breast cancer. Nature.

[84] Shankar D., Merchand-Reyes G., Buteyn N.J., Santhanam R., Fang H., Kumar K., Mo X., Ganesan L.P., Jarjour W., Butchar J.P. (2023). Inhibition of BET Proteins Regulates Fcγ Receptor Function and Reduces Inflammation in Rheumatoid Arthritis. Int. J. Mol. Sci..

[85] Duan Q., McMahon S., Anand P., Shah H., Thomas S., Salunga H.T., Huang Y., Zhang R., Sahadevan A., Lemieux M.E. (2017). BET bromodomain inhibition suppresses innate inflammatory and profibrotic transcriptional networks in heart failure. Sci. Transl. Med..

[86] Borck P.C., Guo L.-W., Plutzky J. (2020). BET Epigenetic Reader Proteins in Cardiovascular Transcriptional Programs. Circ. Res..

[87] Tsujikawa L.M., Fu L., Das S., Halliday C., Rakai B.D., Stotz S.C., Sarsons C.D., Gilham D., Daze E., Wasiak S. (2019). Apabetalone (RVX-208) reduces vascular inflammation in vitro and in CVD patients by a BET-dependent epigenetic mechanism. Clin. Epigenetics.

[88] Sun Y., Xie Y., Du L., Sun J., Liu Z. (2018). Inhibition of BRD4 attenuates cardiomyocyte apoptosis via NF-κB pathway in a rat model of myocardial infarction. Cardiovasc. Ther..

[89] Padmanabhan A., Alexanian M., Linares-Saldana R., González-Terán B., Andreoletti G., Huang Y., Connolly A.J., Kim W., Hsu A., Duan Q. (2020). BRD4 (Bromodomain-Containing Protein 4) Interacts with GATA4 (GATA Binding Protein 4) to Govern Mitochondrial Homeostasis in Adult Cardiomyocytes. Circulation.

[90] Shahid S., Pantakani M., Binder L., Fischer A., Pantakani K., Asif A.R. (2022). Small Molecule BRD4 Inhibitors Apabetalone and JQ1 Rescues Endothelial Cells Dysfunction, Protects Monolayer Integrity and Reduces Midkine Expression. Molecules.

[91] Mohammed S.A., Albiero M., Ambrosini S., Gorica E., Karsai G., Caravaggi C.M., Masi S., Camici G.G., Wenzl F.A., Calderone V. (2022). The BET Protein Inhibitor Apabetalone Rescues Diabetes-Induced Impairment of Angiogenic Response by Epigenetic Regulation of Thrombospondin-1. Antioxid. Redox Signal..

[92] Tsujikawa L.M., Kharenko O.A., Stotz S.C., Rakai B.D., Sarsons C.D., Gilham D., Wasiak S., Fu L., Sweeney M., Johansson J.O. (2022). Breaking boundaries: Pan BETi disrupt 3D chromatin structure, BD2-selective BETi are strictly epigenetic transcriptional regulators. Biomed. Pharmacother..

[93] Piquereau J., Boet A., Péchoux C., Antigny F., Lambert M., Gressette M., Ranchoux B., Gambaryan N., Domergue V., Mumby S. (2019). The BET Bromodomain Inhibitor I-BET-151 Induces Structural and Functional Alterations of the Heart Mitochondria in Healthy Male Mice and Rats. Int. J. Mol. Sci..

[94] Sun J., Gui Y., Zhou S., Zheng X.-L. (2023). Unlocking the secrets of aging: Epigenetic reader BRD4 as the target to combatting aging-related diseases. J. Adv. Res..

[95] Napoli C., Bontempo P., Palmieri V., Coscioni E., Maiello C., Donatelli F., Benincasa G. (2021). Epigenetic Therapies for Heart Failure: Current Insights and Future Potential. Vasc. Health Risk Manag..

[96] Ruscica M., Corsini A., Ferri N., Banach M., Sirtori C.R. (2020). Clinical approach to the inflammatory etiology of cardiovascular diseases. Pharmacol. Res..

[97] Ray K.K., Nicholls S.J., Buhr K.A., Ginsberg H.N., Johansson J.O., Kalantar-Zadeh K., Kulikowski E., Toth P.P., Wong N., Sweeney M. (2020). Effect of Apabetalone Added to Standard Therapy on Major Adverse Cardiovascular Events in Patients with Recent Acute Coronary Syndrome and Type 2 Diabetes: A Randomized Clinical Trial. JAMA—J. Am. Med. Assoc..

[98] Mills R.J., Humphrey S.J., Fortuna P.R.J., Lor M., Foster S.R., Quaife-Ryan G.A., Johnston R.L., Dumenil T., Bishop C., Rudraraju R. (2021). BET inhibition blocks inflammation-induced cardiac dysfunction and SARS-CoV-2 infection. Cell.

[99] McHugh D., Gil J. (2018). Senescence and aging: Causes, consequences, and therapeutic avenues. J. Cell Biol..

[100] Chen M.S., Lee R.T., Garbern J.C. (2022). Senescence mechanisms and targets in the heart. Cardiovasc. Res..

[101] Owens W.A., Walaszczyk A., Spyridopoulos I., Dookun E., Richardson G.D. (2021). Senescence and senolytics in cardiovascular disease: Promise and potential pitfalls. Mech. Ageing Dev..

[102] Bernadotte A., Mikhelson V.M., Spivak I.M. (2016). Markers of cellular senescence. Telomere shortening as a marker of cellular senescence. Aging.

[103] Canale P., Campolo J., Borghini A., Andreassi M.G. (2023). Long Telomeric Repeat-Containing RNA (TERRA): Biological Functions and Challenges in Vascular Aging and Disease. Biomedicines.

[104] Ogrodnik M. (2021). Cellular aging beyond cellular senescence: Markers of senescence prior to cell cycle arrest in vitro and in vivo. Aging Cell.

[105] Tang X., Li P.-H., Chen H.-Z. (2020). Cardiomyocyte Senescence and Cellular Communications within Myocardial Microenvironments. Front. Endocrinol..

[106] Zheng X., Diktonaite K., Qiu H. (2023). Epigenetic Reader Bromodomain-Containing Protein 4 in Aging-Related Vascular Pathologies and Diseases: Molecular Basis, Functional Relevance, and Clinical Potential. Biomolecules.

[107] Lewis-McDougall F.C., Ruchaya P.J., Domenjo-Vila E., Shin Teoh T., Prata L., Cottle B.J., Clark J.E., Punjabi P.P., Awad W., Torella D. (2019). Aged-senescent cells contribute to impaired heart regeneration. Aging Cell.

[108] Marino F., Scalise M., Salerno N., Salerno L., Molinaro C., Cappetta D., Torella M., Foti D., Sasso F.C., Mastroroberto P. (2022). Diabetes-Induced Cellular Senescence and Senescence-Associated Secretory Phenotype Impair Cardiac Regeneration and Function Independently of Age. Diabetes.

[109] Li X., Liu J., Lu Q., Ren D., Sun X., Rousselle T., Tan Y., Li J. (2019). AMPK: A therapeutic target of heart failure-not only metabolism regulation. Biosci. Rep..

[110] Nehlin J.O. (2023). Senolytic and senomorphic interventions to defy senescence-associated mitochondrial dysfunction. Adv. Protein Chem. Struct. Biol..

[111] Belakova B., Wedige N.K., Awad E.M., Hess S., Oszwald A., Fellner M., Khan S.Y., Resch U., Lipovac M., Šmejkal K. (2023). Lipophilic Statins Eliminate Senescent Endothelial Cells by inducing Anoikis-Related Cell Death. Cells.

[112] Belcastro E., Rehman A.U., Remila L., Park S.-H., Gong D.S., Anton N., Auger C., Lefebvre O., Goetz J.G., Collot M. (2021). Fluorescent nanocarriers targeting VCAM-1 for early detection of senescent endothelial cells. Nanomedicine.

[113] Thapa R.K., Nguyen H.T., Jeong J.-H., Kim J.R., Choi H.-G., Yong C.S., Kim J.O. (2017). Progressive slowdown/prevention of cellular senescence by CD9-targeted delivery of rapamycin using lactose-wrapped calcium carbonate nanoparticles. Sci. Rep..

[114] Di Micco R., Krizhanovsky V., Baker D., d’Adda di Fagagna F. (2021). Cellular senescence in ageing: From mechanisms to therapeutic opportunities. Nat. Rev. Mol. Cell Biol..

[115] Sardu C., Onofrio N.D., Torella M., Portoghese M., Mureddu S., Loreni F., Ferraraccio F., Panarese I., Trotta M.C., Gatta G. (2021). Metformin Therapy Effects on the Expression of Pericoronary Fat Excised from Pre-Diabetic Patients with Acute Myocardial Infarction. Biomedicines.

[116] Onofrio N.D., Sardu C., Trotta M.C., Scisciola L., Turriziani F., Ferraraccio F., Panarese I., Petrella L., Fanelli M., Modugno P. (2021). Sodium-glucose co-transporter2 expression and in fl ammatory activity in diabetic atherosclerotic plaques: Effects of sodium-glucose co-transporter2 inhibitor treatment. Mol. Metab..

[117] Nesti L., Pugliese N.R., Sciuto P., Natali A. (2020). Type 2 diabetes and reduced exercise tolerance: A review of the literature through an integrated physiology approach. Cardiovasc. Diabetol..

[118] Guazzi M., Bandera F., Ozemek C., Systrom D., Arena R. (2017). Cardiopulmonary Exercise Testing What Is its Value?. J. Am. Coll. Cardiol..

[119] Sweeney M., Cook S.A., Gil J. (2023). Therapeutic opportunities for senolysis in cardiovascular disease. FEBS J..

